# Understanding Phenotypical Character Evolution in Parmelioid Lichenized Fungi (Parmeliaceae, Ascomycota)

**DOI:** 10.1371/journal.pone.0083115

**Published:** 2013-11-29

**Authors:** Pradeep K. Divakar, Frank Kauff, Ana Crespo, Steven D. Leavitt, H. Thorsten Lumbsch

**Affiliations:** 1 Departamento de Biología Vegetal II, Facultad de Farmacia, Universidad Complutense de Madrid, Madrid, Spain; 2 FB Biologie, Molecular Phylogenetics, TU Kaiserslautern, Kaiserslautern, Germany; 3 Science & Education, The Field Museum, Chicago, Illinois, United States of America; Georg-August-University of Göttingen Institute of Microbiology & Genetics, Germany

## Abstract

Parmelioid lichens form a species-rich group of predominantly foliose and fruticose lichenized fungi encompassing a broad range of morphological and chemical diversity. Using a multilocus approach, we reconstructed a phylogeny including 323 OTUs of parmelioid lichens and employed ancestral character reconstruction methods to understand the phenotypical evolution within this speciose group of lichen-forming fungi. Specifically, we were interested in the evolution of growth form, epicortex structure, and cortical chemistry. Since previous studies have shown that results may differ depending on the reconstruction method used, here we employed both maximum-parsimony and maximum-likelihood approaches to reconstruct ancestral character states. We have also implemented binary and multistate coding of characters and performed parallel analyses with both coding types to assess for potential coding-based biases. We reconstructed the ancestral states for nine well-supported major clades in the parmelioid group, two higher-level sister groups and the ancestral character state for all parmelioid lichens. We found that different methods for coding phenotypical characters and different ancestral character state reconstruction methods mostly resulted in identical reconstructions but yield conflicting inferences of ancestral states, in some cases. However, we found support for the ancestor of parmelioid lichens having been a foliose lichen with a non-pored epicortex and pseudocyphellae. Our data suggest that some traits exhibit patterns of evolution consistent with adaptive radiation.

## Introduction

Molecular data have revolutionized our understanding of evolution, especially in groups with relatively simple morphology and high levels of phenotypic homoplasy, such as fungi. The enormous increase in knowledge has led to a revival of fungal systematics. This is especially true in fungal groups, including lichen-forming fungi, for which vegetative characters have traditionally played an important role in classification and inferring relationships. Lichenized fungi usually form persistent thalli to house their photosynthetic partner and produce a wide array of secondary metabolites that play a variety of roles in the maintenance of the symbiotic association [1-5]. These phenotypical characters have been heavily utilized in traditional classifications of these organisms [6,7]. However, before molecular data became readily available, evolutionary hypotheses of these important phenotypical characters were largely left to speculation [1-5,8]. Now, in addition to elucidating phylogenetic relationships among fungi, molecular phylogenies are increasingly popular in studying trait evolution, with numerous examples from lichenized fungi providing novel insight into the evolution of chemical and morphological diversity [9-21]. 

Although ancestral character state reconstruction approaches provide powerful tools for assessing trait evolution, the methods are not without problems. Ekman and co-workers [22] pointed out a number of limitations in the practical application of these methods. Among these, most methods assume a constant rate of character change throughout the evolutionary history, an assumption that is probably extremely rare, if present at all. Further, it has been shown that reconstructions of ancestral states may be biased by neglecting the effect of the character states on diversification rates [23]. Maximum likelihood approaches for reconstructing ancestral character states mostly use branch lengths to estimate the probability of character change. However, this assumes that there is a correlation of rate of substitution change and rate of phenotypical change. Such a correlation has been reported [24], but has been questioned by others [25-27]. In addition to these problems, the coding of characters, either binary or multistate, may affect the outcome of ancestral character state reconstructions [28,29]. Given the various assumptions and methodological differences associated with different ancestral character state reconstruction methods, it is not surprising that different methods may yield diverging results [22,30]. Hence, we used both maximum parsimony- and ML-based approaches to accommodate for potential influence of branch lengths on the ancestral character state reconstructions, in addition to the potential effect of different character coding approaches. 

Parmelioid lichens constitute the largest clade within the speciose family Parmeliaceae [17,31]. Parmelioid lichens include common and well-known species, some of which are frequently used in biomonitoring atmospheric pollution [32-34]. Over 1800 species have been described in the group [8,35,36]. Recent phylogenetic studies have supported the monophyly of the core group of parmelioid lichens [15,17,31,37,38]. Most parmelioid species form foliose thalli, predominantly rhizinate with laminal apothecia and simple, hyaline ascospores [39]. Parmelioid lichens also include some genera with morphologies deviating from the typical foliose growth form, such as the peltate *Omphalodiella*, subcrustose *Karoowia*, subfruticose *Almbornia*, umbilicate *Xanthomaculina*, and even lichenicolous fungal genus *Nesolechia*, which has been shown to belong to the parmelioid clade [17,31,40]. 

Morphological diversity in parmelioid lichens is not limited to different growth forms, but also includes ultrastructural characters and diverse secondary metabolites. Within the group, two major types of thallus perforations can be found that allow gas exchange of the photosynthetic partner. These include pores in the epicortex and cortical structures that reach the algal layer, which are called pseudocyphellae. The epicortex is a polysaccharide layer about 0.6 μm thick, lying over the cortex [41-44]. Pores are 15–40 μm in diam. Pseudocyphellae have a diam. of 200–2000 μm. The cortex is dissolved or partially reduced in pseudocyphellae, while it is not when pores are developed. The chemical diversity is enormous in the family. Secondary metabolites reported for this family belong to a number of substance classes, including: orcinol and beta-orcinol depsides and depsidones, xanthones, anthraquinones, secalonic acids, amino acid derivatives, pulvinic acid derivatives, benzyl esters, diphenyl ethers, terpenoids, and aliphatic acids [39,45]. Cortical substances play an important role for the lichens in screening UV and visible light [46,47] and anti-herbivory [48].While the majority of these substances are deposited in the algal layer or medulla, three different types of cortical substances are present in parmelioid lichens: atranorin, usnic acid or melanoid pigments [35,39]. 

In order to understand phenotypical character evolution in parmelioid lichens, we used a previously published molecular phylogeny of parmelioid lichens [31]. Specifically, we trace trait evolution of growth forms, cortical opening, and chemistry in major clades of parmelioid lichens using a data matrix including 323 samples to elucidate the evolution of these phenotypical characters. The data set used here is identical to the 3GENE data set previously published [31] for a phylogenetic classification of parmelioid lichens, which includes only samples for which sequence data for at least three of the four sampled loci are present. We accommodated for phylogenetic uncertainty by using a Bayesian tree sampling. Based on this tree sampling, ancestral character state reconstructions of morphological and chemical characters were estimated using maximum-likelihood and maximum parsimony and used to evaluate previous hypotheses developed on the basis of classical comparative methods. Further, we compared results of different character-coding methods.

## Results

### Phylogenetic analysis

**Figure 1:**
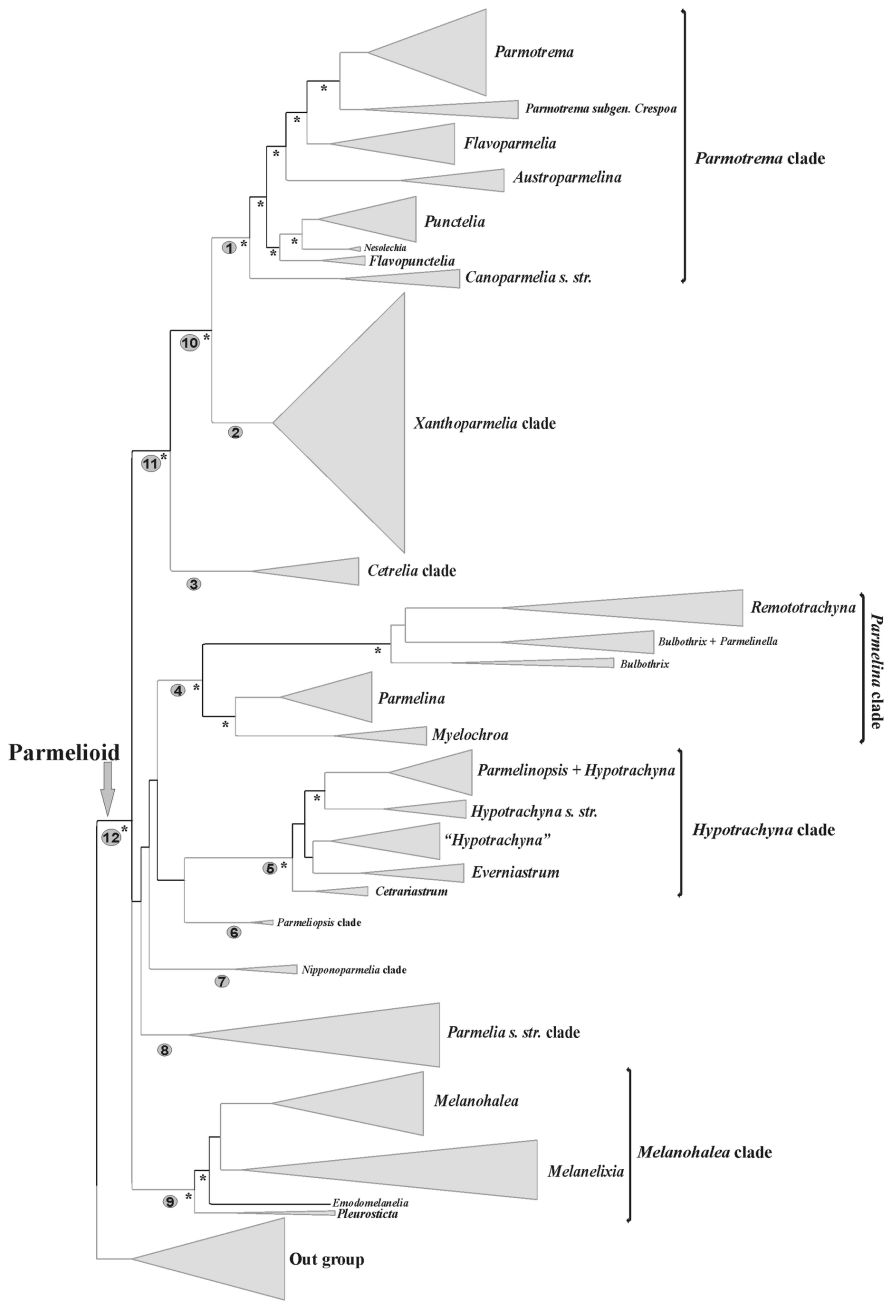
Cartoon tree showing nine major clades of parmelioid lichens identified in [31], the well supported higher-level sister groups *Parmotrema+Xanthoparmelia* clade, Cetrelia+Parmotrema*+Xanthoparmelia* clade, and the entire parmelioid group. Nodes indicated as 1-12 were used for ancestral state reconstruction. The cartoon tree is drawn using FigTree using the Bayesian consensus tree of the 3GENE data set of [31]. Asterisks indicate strongly supported nodes, i.e. nodes with ML-bootstrap support of 70% or higher and posterior probabilities of 0.95 or higher.

**Figure 2:**
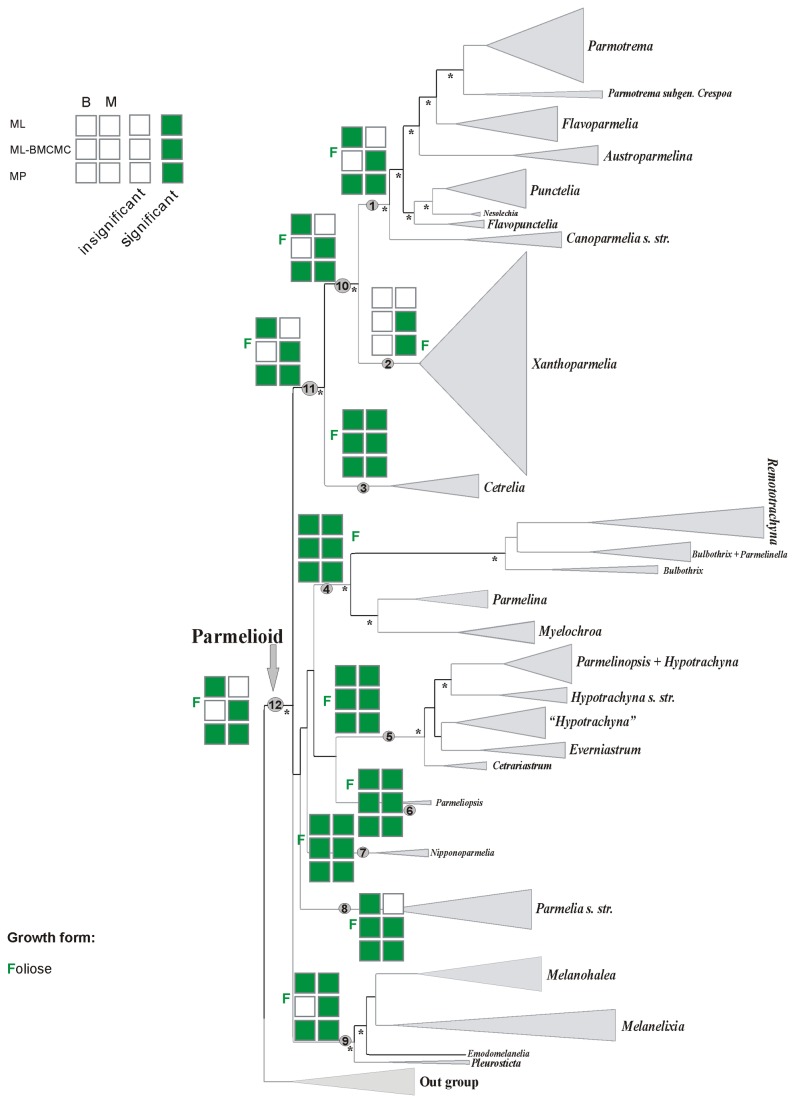
Ancestral character state reconstructions of growth forms in parmelioid lichens. Binary and multistate coding datasets analysed with ML, ML-BMCMC and MP approaches. B=Binary coding, M=Multistate coding, F=Foliose.

**Figure 3:**
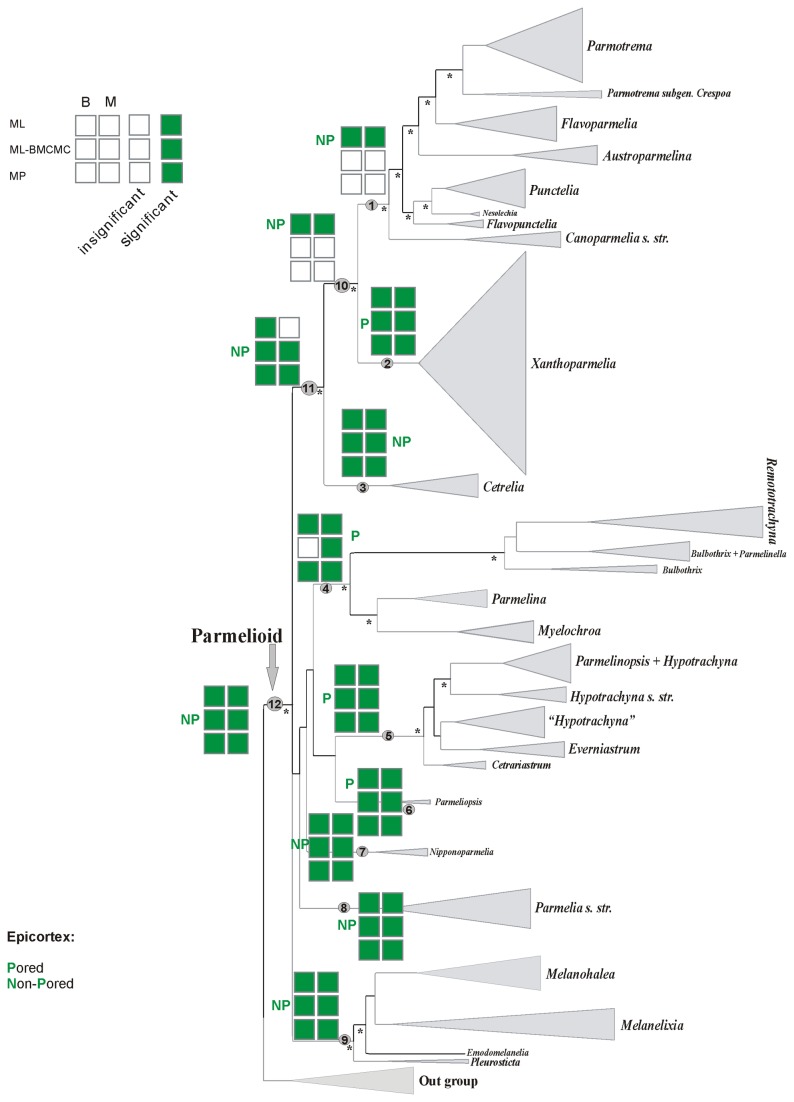
Ancestral character state reconstructions of epicortex structure. Binary and multistate coding datasets analysed with ML, ML-BMCMC and MP approaches. B=Binary coding, M=Multistate coding, P=Pored, NP=Non-pored.

**Figure 4:**
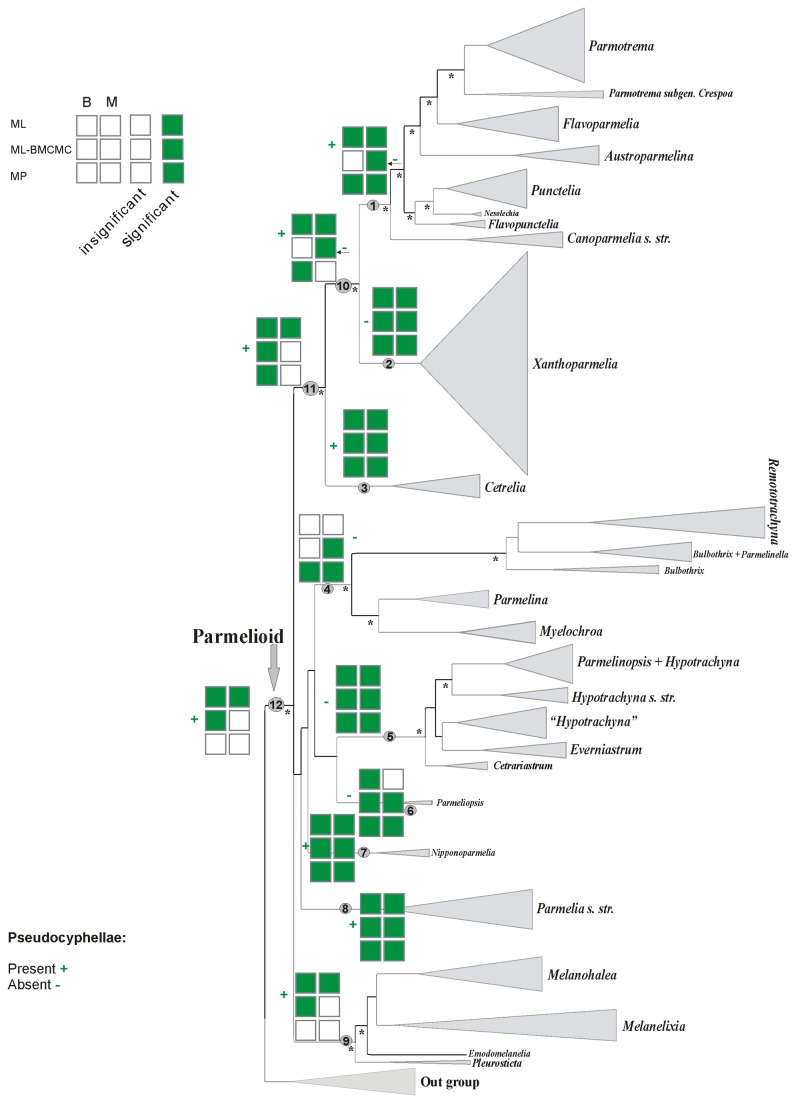
Ancestral character state reconstructions of pseudocyphellae presence or absence. Binary and multistate coding datasets analysed with ML, ML-BMCMC and MP approaches. B=Binary coding, M=Multistate coding, +=Present, -=Absent.

**Figure 5:**
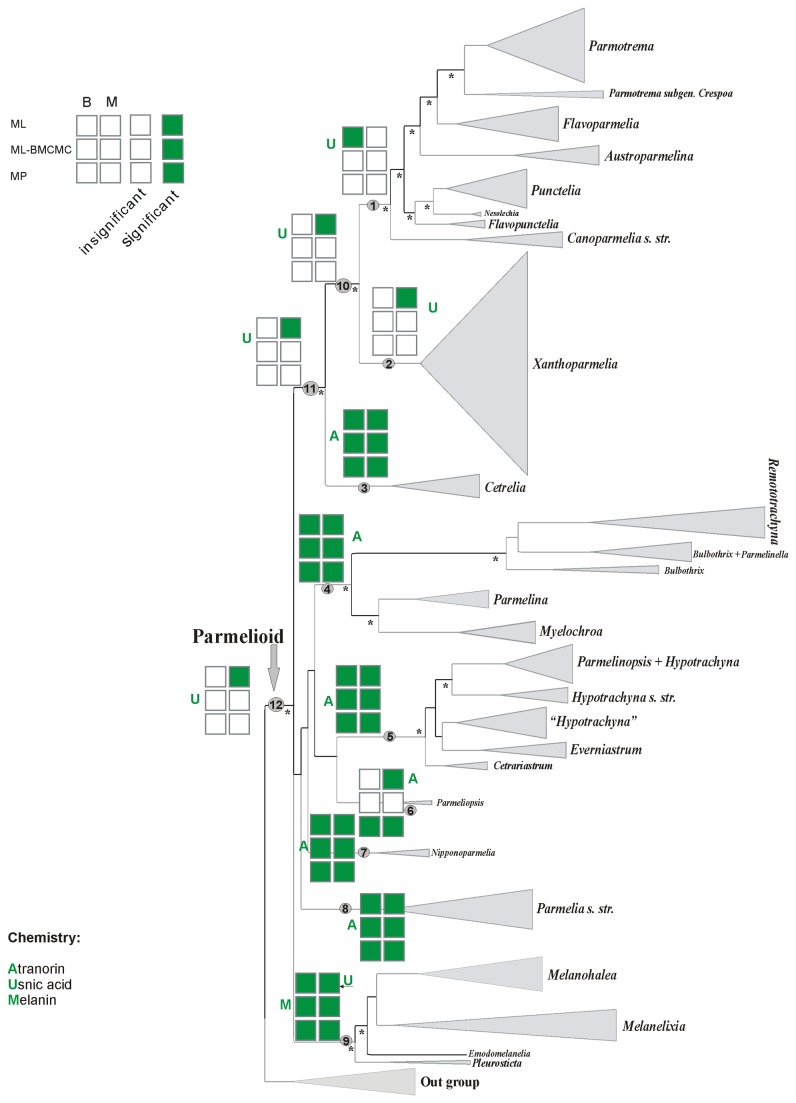
Ancestral character state reconstructions of cortical chemistry. Binary and multistate coding datasets analysed with ML, ML-BMCMC and MP approaches. B=Binary coding, M=Multistate coding, A=Atranorin, U=Usnic acid, M=Melanin.

The phylogenetic analyses of this data set have been published and discussed previously [31] and hence the discussion of the data set, summary statistics, congruence of loci and phylogenetic relationships are not repeated here. We focused our ancestral character reconstruction analyses on the nine major clades of parmelioid lichens identified in [31], the well supported higher-level sister groups *Parmotrema+Xanthoparmelia* clade, *Cetrelia+Parmotrema+Xanthoparmelia* clade, and the entire parmelioid group (indicated as nodes 1-12 in Figure 1). The nine major clades and their phenotypical characters are discussed in detail in [31]. The character states of each trait examined here for each clade are listed in Table 1. Figures 2-5 and tables S1 and S2 summarize the results of the different ancestral character reconstructions with strongly supported results under maximum parsimony –MP–, maximum likelihood reconstruction over BMCMC trees using Mesquite–ML-BMCMC-, and maximum likelihood reconstruction with BayesTraits–ML–. Below, we describe the results of the ancestral character reconstructions and focus on the nodes that received support in at least one of the analyses (see 31).

**Table 1:** Character states of each trait analyzed.

No.	Character states	Trait
1	Growth forms	Crustose
		Foliose
		Fruticose
		Subcrustose
		Umbilicate
2	Epicortex structure	Non-Pored
		Pored
3	Pseudocyphellae	Present
		Absent
4	Cortical chemistry	Atranorin
		Melanin
		Usnic acid

### Testing for restricted vs. unrestricted models of character change

When comparing restricted vs. unrestricted models for the multistate character coding, the difference was always greater than 10 log likelihood units, and thus the unrestricted model was chosen for all subsequent analyses. For the binary coding, in several characters (umbilicate growth, presence of epicortex, atranorin) the difference was smaller than 2 units of likelihood. However, in order to efficiently process the large number of analyses and results, we decided to use unrestricted models for all analyses.

The results of the ancestral character state reconstructions for binary and multistate coding and for the three types of analysis are summarized in Figures 2-5 and discussed below. Detailed results are given in tables S1-S2. 

### Ancestral character state reconstructions of the growth form

Results of ancestral character state reconstructions of the following four growth forms, foliose, subcrustose, crustose and umbilicate are depicted in Figure 2. Differences among the ancestral state reconstruction of the growth form were found for clades 2, 10, 11, and the basal node 12. All reconstruction methods estimated foliose growth as ancestral state for the remaining clades. However, node 2 was only supported by multistate characters using maximum likelihood and maximum likelihood using posterior Bayesian trees. A foliose growth form was reconstructed as ancestral for the entire parmelioid clade (node 12) and the *Cetrelia+Parmotrema+Xanthoparmelia* clade (node 11) in the binary-coded MP and ML, and multistate-coded MP and ML-BMCMC analyses. A foliose growth form received significant support in the binary- and multistate-coded MP, binary- ML, and multistate-coded ML-BMCMC analyses for the ancestral node of the *Parmotrema+Xanthoparmelia* clade (node 10) but lacked significant support in the binary ML-BMCMC and multistate ML analyses. For the *Parmotrema* clade (node 1) foliose growth form received strong support in both binary- and multistate-coded MP reconstructions, the binary-coded ML analysis, and multistate-coded of ML-BMCMC analyses. A foliose ancestral state was also inferred in the binary-coded ML-BMCMC and multistate-coded ML reconstructions, although it lacked support (see Figure 2). The ancestral character state of the *Xanthoparmelia* clade (node 2) was reconstructed as having a foliose growth form in the MP and ML-BMCMC method with significant support using multistate-coding method. However, an ancestral foliose growth form for *Xanthoparmelia* lacked significant support in the binary-coded MP and ML-BMCMC-based methods, and in ML analyses using both coding methods. A foliose growth form was reconstructed as ancestral for the *Cetrelia* clade (node 3) in all the analyses using both coding methods. The ancestral growth form for node basal to the *Parmelina* clade (node 4) was reconstructed as a foliose, with significant result in both coding MP, ML and ML-BMCMC analyses. For node 5 (base of *Hypotrachyna* clade) all reconstruction methods significantly inferred a foliose growth form as the ancestral state. They also yielded significant support for a foliose growth form at node 6, which included the genus *Parmeliopsis*. All analyses estimated a foliose growth form as ancestral for the *Nipponoparmelia* clade (node 7) with significant support. A foliose growth form was also reconstructed for the ancestor of the *Parmelia* clade (node 8) with significant support by all methods, with the exception of the multistate-coded ML reconstruction, which lacked support. The MP and ML methods using both coding analyses, and multistate-coded ML-BMCMC estimated the ancestral character state for the growth form of the *Melanohalea* clade (node 9) as being foliose, while the binary-coded ML-BMCMC analysis did not yield significant results. 

### Ancestral character state reconstructions of the epicortical structure and pseudocyphellae

Results of the reconstructions of epicortical structure are presented in Figures 3 and 4. Two morphological traits of the epicortex structure were analysed: (1) the presence of pores and (2) the presence of pseudocyphellae. All analyses estimated with significant support the basal state for the parmelioid clade (node 12) as having a non-pored epicortex (Figure 3). An ancestral form having pseudocyphellae was inferred in the ML analyses using both coding methods and in the binary-coded ML-BMCMC reconstruction. However, this ancestral character state reconstruction lacked significant support in MP and multistate-coded ML-BMCMC analysis (Figure 4). 

The ancestral character state for the *Cetrelia+Parmotrema+Xanthoparmelia* clade (node 11) was inferred as having a non-pored epicortex with significant support in both MP, ML-BMCMC and binary-coded ML reconstructions, but it lacked support in the multistate-coded ML analysis (Figure 3). Presence of pseudocyphellae was estimated as the ancestral state with both coding methods in the ML analyses and the binary-coded method in the ML-BMCMC and MP reconstructions. The presence of pseudocyphellae as the ancestral character state lacked support in multistate-coded MP and ML-BMCMC reconstructions (Figure 4). 

The results of the ancestral character state reconstructions for the *Parmotrema+Xanthoparmelia* clade (node 10) were less clear (Figures 3 and 4). While both coding methods in the ML analyses and the binary-coded MP analysis inferred the presence of pseudocyphellae as ancestral state for this clade with significant support (Figure 4), the analysis of the presence of absence of pores did not reveal significant results for both coding methods in the ML-BMCMC and MP analyses (Figure 3). Both coding methods in the ML-based reconstructions gave significant support for the absence of pores (Figure 3). Presence of pseudocyphellae lacked support in the multistate-coded MP and the binary-coded ML-BMCMC reconstructions, while the absence of pseudocyphellae was reconstructed in multistate-coded ML-BMCMC analysis. The ML reconstruction based on binary coding suggested the absence of pores with significant support for the base of the *Parmotrema* clade (node 1). (Figure 3). The ML and MP reconstructions, however, estimated consistently for node 1 the presence of pseudocyphellae with significant support. In contrast, the multistate-coded ML-BMCMC reconstruction estimated significant support for the absence of pseudocyphellae, although the absence of pseudocyphellae lacked support in the binary-coded ML-BMCMC analysis (Figure 4). 

Ancestral character state reconstructions of the epicortex structure and pseudocyphellae were largely consistent among methods for the clades discussed below. The presence of a pored epicortex and absence of pseudocyphellae was inferred for the ancestor of the *Xanthoparmelia* clade (node 2) (Figures 3 and 4). For the base of *Cetrelia* clade (node 3), a non-pored epicortex and the presence of pseudocyphellae were reconstructed with very significant support in all analyses (Figures 3, 4). For the *Parmelina* clade (node 4), a pored epicortex was consistently estimated in all analyses, except binary-coded ML-BMCMC where it lacked support. However, the absence of pseudocyphellae was only reconstructed in the MP and multistate-coded ML-BMCMC analyses (Figure 4). For the ancestor of the clades *Hypotrachyna* (node 5) and *Parmelinopsis* (node 6), a pored epicortex and the absence of pseudocyphellae were estimated in all analyses (Figures 3, 4). A non-pored epicortex and pseudocyphellae were reconstructed for the base of *Nipponoparmelia* (node 7) and *Parmelia* (node 8) clades in all analyses (Figures 3, 4). For the base of the *Melanohalea* clade (node 9), a non-pored epicortex and the presence of pseudocyphellae was reconstructed in ML and binary-coded ML-BMCMC analyses with significant support. However, this reconstruction lacked support in the MP and multistate-coded ML-BMCMC analyses (Figures 3, 4).

### Ancestral character state reconstructions of the cortical chemistry

Results of ancestral character state estimations for cortical chemistry are shown in Figure 5. For the entire parmelioid clade (node 12) the multistate-coded ML analysis estimated usnic acid as the ancestral character, while the binary-coded ML and both coding methods in the MP and ML-BMCMC analyses did not reveal significant results. For the *Cetrelia+Parmotrema+Xanthoparmelia* clade (node 11) and the *Parmotrema+Xanthoparmelia* clade (node 10), the multistate-coded ML analysis inferred usnic acid as the ancestral character, while the other analyses did not reveal significant results (Figure 5). For node 1 (*Parmotrema* clade) the binary-coded ML analysis estimated the presence of usnic acid as ancestral character with significant support (Figure 5). However, the other analyses did not provide significant support for this ancestral character state. For the base of the *Xanthoparmelia* clade (node 2), the multistate-coded ML analysis recovered usnic acid as the ancestral character state with significant support, while the other analyses did not significantly support this ancestral character state (Figure 5). All reconstruction methods reconstructed atranorin as the ancestral state for the *Cetrelia* clade (node 3), the *Parmelina* clade (node 4), and the *Hypotrachyna* clade (node 5) with significant support (Figure 5). The multistate-coded ML and the binary-MP and multistate-MP analyses revealed significant support for the presence of atranorin at the base of *Parmeliopsis* (node 6), while the other methods did not support for any character state with statistical significance (Figure 5). All reconstruction methods estimated atranorin as the ancestral state for the *Nipponoparmelia* (node 7) and *Parmelia* (node 8) clades with significant support. For the *Melanohalea* clade (node 9), melanin was reconstructed as ancestral character state in both coding methods in the MP, ML-BMCMC analyses and the binary-coded ML with significant support. In contrast, usnic acid was inferred in the multistate-coded ML analysis with significant support (Figure 5).

## Discussion

Ancestral character state reconstruction methods are widely used to understand evolutionary histories for a broad spectrum of organisms [13,14,25,49-53] and our analyses resulted in identical reconstructions, regardless of the type of character-coding, with few exceptions. However, we show that different coding strategies and reconstruction methods can yield conflicting reconstructions of ancestral character states. In addition, we show that methods explicitly taking branch lengths into account (i.e. ML methods) do not necessarily result in more similar estimates than those methods that do not include branch lengths (i.e. MP). Our work adds to previous studies suggesting that caution must be taken when inferring the ancestral state of a character, and a variety of methods should be used to mitigate potential methodological-biases [22]. 

### Differences among character reconstruction methods

In this study, all reconstruction methods estimated foliose growth as ancestral state for most clades. This is not surprising, given that the large majority of parmelioid lichens are foliose and that the highest growth form diversity, including subcrustose, fruticose, peltate and umbilicate species, is restricted to the Xanthoparmelia clade (see node 2 in Figure 2) [31,39,54,55]. Both MP and ML methods reconstructed foliose growth form as ancestral for all four clades but only in part with significant support. 

In parmelioid lichens, the presence or absence of pseudocyphellae and pores in the epicortex has frequently been used to circumscribe lineages [39,42,44]. In agreement with a previous study [15], all ancestral character state reconstruction methods estimated a non-pored epicortex with pseudocyphellae as ancestral character state for the basal node of parmelioid lichens (12), largely with significant support (presence of pseudocyphellae failed to receive significant support in MP and one ML reconstruction) (Figures 3, 4). While for most nodes, different reconstruction methods yielded the same ancestral node reconstructions (with or without support), we observed a difference between different character coding methods in one ML-based reconstruction analysis for the reconstruction of presence or absence of pseudocyphellae for two nodes (see below). 

Secondary metabolites traditionally play an important role in taxonomy of lichenized ascomycetes [2,56] and cortical chemistry is widely used to circumscribe genera in parmelioid lichens [31,39,57]. In this study, reconstruction methods yielded generally consistent results (Figure 5), with the exception of node 9 (the Melanohalea clade) for which most methods reconstructed melanin as ancestral character state, while one ML method reconstructed usnic acid with significant support. 

### Differences between binary and multistate-coding

Previous studies on fungi have shown different results in ancestral character state reconstruction between binary and multistate-coding procedures [28,29]. We also compared the two coding methods with our data set. In our analyses, most reconstructions were identical, regardless the type of character-coding method, with few exceptions. None of the contradicting results included MP reconstructions. For ML reconstructions, the pseudocyphellae at nodes 1 and 10 were reconstructed as present using binary and absent using multistate-coding. However, the former reconstructions did not receive support and only for the chemistry at node 9 a conflict was found in the ML reconstructions with significant support for melanin using binary and usnic acid using multistate-coding. These results indicate that MP and ML reconstruction methods are generally resilient to different character coding approaches.

### An evolutionary scenario of character evolution in parmelioid lichens

Parmelioid lichens are one of the most species-rich groups in lichen-forming fungi [8,31,36]. Disparities in species richness among clades are often explained by the evolution of key innovative traits, which lead to adaptive radiation [58-60]. These features affect the lineage diversification rates and are expected to leave an imprint in the phylogeny of the affected group [61]. Our previous study has shown that parmelioid lichens evolved near the Cretaceous–Tertiary boundary (60 Ma.) and lineages within parmelioid lichens diversified at different times during the Eocene, Oligocene, Miocene and early Pliocene in coincidence with periods of cooling of the climate [62-65]. In this study, we find support for the ancestor of parmelioid lichens having been a foliose lichen with a non-pored epicortex and pseudocyphellae. Whether or not the ancestor contained usnic acid remains uncertain. Subcrustose and umbilicate growth forms have evolved independently from foliose species in few lineages. Our data indicate that parmelioid lichens with a pored epicortex and lacking pseudocyphellae have also evolved independently several times, suggesting that there is an adaptive value of this type of cortex. High levels of trait changes during the diversification processes are often linked with adaptive radiation [66]. Further, shifting of growth forms has been shown to be implicated in adaptive radiation in other groups, such as angiosperm lineages [66,67]. In other organisms, some lineages appear to possess key innovative traits that promote radiation, such as those associated with pollinator specificity in plants [68,69] and sexual selection in animals [70,71]. We here hypothesize that a foliose thallus with a non-pored epicortex and pseudocyphellae – key innovative traits –has played a predominant role in habitat colonization shortly after the Cretaceous–Tertiary boundary. Pseudocyphellae have been shown to enhance gas exchange [72,73] and to be correlated with colder climates [74].

Parmelioid lichens and lichens in general produce a broad spectrum of secondary metabolites and polysaccharides. Many of these, especially cortical substances have important ecological functions; such as protection from solar UV radiation and as defence against herbivores [75-77], but secondary metabolites also possess a variety of biological activities, including antibiotic, antiviral, antipyretic, and cytostatic effects [78]. Thus, secondary metabolites can be considered as defence traits for species diversification in lichen-forming fungi. In angiosperms, latex has been considered as a key defensive innovation correlated with adaptive radiations [66,79,80]. Within parmelioid lichens there are three main cortical substances: atranorin, melanin and usnic acid. Atranorin results in a grey-coloured upper surface, while usnic acid provides yellow-green coloured upper surfaces, and melanin results in brown to olive-brown thalli. Our data suggest that cortical substances appear as key innovative traits and high level of trait change during the diversification promotes adaptive radiation in parmelioid occupying different climatic regions.

Usnic acid appears as ancestral traits for parmelioid lineage as whole and also for inner clades as Parmotrema clade, Parmotrema+Xanthoparmelia clade, Cetrelia+Parmotrema+Xanthoparmelia clade and the Xanthoparmelia clade, although most reconstruction analyses lacked statistical support. In addition to usnic acid, some taxa in the Xanthoparmelia clade also, or instead, contain atranorin and melanin as cortical substances [8,31,36]. Additional studies focusing on the evolution of secondary metabolites in this group are needed. The species-rich genus Xanthoparmelia (ca. 800 species) has its centre of distribution in arid to sub-arid regions (e.g. in Australia, South Africa). Within parmelioid lichens, this is the only clade that is species-rich under such climatic conditions. The Xanthoparmelia clade includes a number of short branches along the phylogeny and has slow substitution rates, suggesting rapid radiations [38,54,81]. Atranorin was recovered as the ancestral cortical chemistry state - with strong support in all analyses - for the Cetrelia clade, Hypotrachyna clade, Nipponoparmelia clade, Parmelia clade and Parmelina clade. The Hypotrachyna clade is another speciose group, including ca. 250 species and shows accelerated substitution rates [15,38,57,82]. Species included in this clade contain atranorin, lichexanthone and usnic acid, and are widely distributed in mountains in humid tropical to sub-tropical regions. Melanin was recovered as the ancestral state for the Melanohalea clade in all but one analysis. Species included in Melanohalea clade are largely distributed to boreal regions and our results suggest that melanin promotes adaptive radiation in this lineage. 

Concluding, we have used an approach including different reconstruction methods to increase confidence in the ancestral character reconstructions and based on this we developed an evolutionary scenario of phenotypical evolution in parmelioid lichens. We have shown here that some traits exhibit patterns of evolution consistent with an impact on adaptive radiation and that characters are not randomly distributed over the phylogenetic tree.

## Materials and Methods

### Characters state reconstruction

The 3GENE – four locus – tree from [31] was used as topology for all ancestral state reconstructions. Characters for the analysis (growth form, chemistry, epicortex, and pseudocyphellae) were analyzed using both binary and multistate character state coding (for the detailed coding schemes see tables S1-S2). 

### Testing for restricted vs. unrestricted models of character change

Ancestral state reconstructions can be carried out using unrestricted models, where the rates for the transitions between two states are allowed to differ, or with restricted models, where all rates are assumed to be equal. We used BayesTraits for Linux v1.0 (http://www.evolution.reading.ac.uk/BayesTraits.html) in order to test whether unrestricted models perform significantly better than restricted models [83]. Using the 'restrict' command of BayesTraits, log likelihoods of restricted and unrestricted models were compared for each character. Differences in log likelihood greater than 2 are generally considered significant. 

### Ancestral state reconstruction with BayesTraits using Maximum Likelihood (ML)

For each analyzed node and each character, the ancestral state for this node was successively fixed to each of the possible states (e. g. 0/1 for binary coding) using the 'fossil' command of BayesTraits. Using an unrestricted model of character change, the maximum likelihood for the tree with the ancestral state fixed at this node was estimated and the difference in log likelihood calculated. A difference of 2 log likelihood units was considered significant evidence [84].

### Maximum Likelihood reconstruction over a sample of BMCMC trees using Mesquite (ML-BMCMC)

In order to include topological uncertainty into the ancestral state reconstruction, we used the “Trace character over trees” method of Mesquite. 1000 trees were randomly sampled from the post-burnin of the 3GENE Bayesian analysis of [31], and an ML reconstruction was carried out for each individual tree using an unrestricted (2-par in Mesquite) model of character evolution. Mesquite displays a summary for the probability for each node and each character, indicating the probability for the different states, and also taking into account ambiguous reconstructions and the percentage of Bayesian trees in which the given node was not present (tables S1-S2).

### Ancestral state reconstruction with Mesquite using Maximum Parsimony (MP)

In contrast to likelihood and Bayesian methods, maximum parsimony does not take into account branch lengths when reconstructing ancestral states. We used Mesquite 2.75 [85] to carry out an MP reconstruction for the multistate and binary character data sets. The reconstructions were performed over the same 1000 randomly sampled trees from the post-burnin of the 3GENE Bayesian analysis of [31] as in the ML analyses. 

## Supporting Information

Table S1 **Results of the different reconstruction methods for parmelioid lichens at selected nodes.** Shown are ln likelihood differences (ML, BayesTraits), percentage of trees for which the character state has been significantly reconstructed (ML-BMCMC, MP, both Mesquite). For ML-BMCMC and MP, values missing to reach a total of 1.00 represent trees for which the node was not present in the set of source trees used for the reconstruction. (see Materials and Methods for details). All analyses were performed using the binary coding (0= absent, 1=present) data set. For MP probabilities only unequivocal reconstructions were counted. For reconstructed nodes see Figure1.
(DOCX)

Table S2 **Results of the different reconstruction methods for parmelioid lichens at selected nodes.** Shown are ln likelihood differences (ML, BayesTraits), percentage of trees for which the character state has been significantly reconstructed (ML-BMCMC, MP, both Mesquite). For ML-BMCMC and MP, values missing to reach a total of 1.00 represent trees for which the node was not present in the set of source trees used for the reconstruction. (see Materials and Methods for details). All analyses were performed with the multistate coding state data set (i.e. 0,1,2,3,4). For MP probabilities only unequivocal reconstructions were counted. For reconstructed nodes see Figure 1.
(DOCX)
