# Supplementary material for: Understanding Phenotypical Character Evolution in Parmelioid Lichenized Fungi (Parmeliaceae, Ascomycota)
Source: PLoS One. 2013 Nov 29;8(11):e83115. doi: 10.1371/journal.pone.0083115 (PMC3843734; doi:10.1371/journal.pone.0083115)
Supplement: Table S1 — Results of the different reconstruction methods for parmelioid lichens at selected nodes. Shown are ln likelihood differences (ML, BayesTraits), percentage of trees for which the character state has been significantly reconstructed (ML-BMCMC, MP, both Mesquite). For ML-BMCMC and MP, values missing to reach a total of 1.00 represent trees for which the node was not present in the set of source trees used for the reconstruction. (see Materials and Methods for details). All analyses were performed using the binary coding (0= absent, 1=present) data set. For MP probabilities only unequivocal reconstructions were counted. For reconstructed nodes see Figure1. (DOCX) [file pone.0083115.s001.docx]

**Table S1**. Results of the different reconstruction methods for parmelioid lichens at selected nodes. Shown are ln likelihood differences (ML, BayesTraits), percentage of trees for which the character state has been significantly reconstructed (ML-BMCMC, MP, both Mesquite). For ML-BMCMC and MP, values missing to reach a total of 1.00 represent trees for which the node was not present in the set of source trees used for the reconstruction. (see Materials and Methods for details). All analyses were performed using the binary coding (0= absent, 1=present) data set. For MP probabilities only unequivocal reconstructions were counted. For reconstructed nodes see Fig.1.

| Node | ML | ML-BMCMC | MP |
| --- | --- | --- | --- |
| Character 1, Foliose growth | | | |
| 1 | 1: 2.93 | 1: 0.82 | 1: 1.00 |
| 2 | Ambiguity | 1: 0.57 | 1: 0.93 |
| 3 | 1: 8.82 | 1: 1.00 | 1: 1.00 |
| 4 | 1: 11.25 | 1: 1.00 | 1: 1.00 |
| 5 | 1: 15.89 | 1: 1.00 | 1: 1.00 |
| 6 | 1: 16.38 | 1: 1.00 | 1: 1.00 |
| 7 | 1: 13.30 | 1: 1.00 | 1: 1.00 |
| 8 | 1: 13.33 | 1: 1.00 | 1: 1.00 |
| 9 | 1: 8.61 | 1: 0.94 | 1: 1.00 |
| 10 | 1: 2.31 | 1: 0.75 | 1: 1.00 |
| 11 | 1: 2.35 | 1: 0.80 | 1: 0.97 |
| 12 | 1: 2.32 | 1: 0.85 | 1: 0.98 |
| Character 2, Fruticose growth | | | |
| 1 | 0: 14.52 | 0: 1.00 | 0: 1.00 |
| 2 | Ambiguity | 0: 1.00 | 0: 1.00 |
| 3 | 0: 15.82 | 0: 1.00 | 0: 1.00 |
| 4 | 0: 14.90 | 0: 1.00 | 0: 1.00 |
| 5 | 0: 18.96 | 0: 1.00 | 0: 1.00 |
| 6 | 0: 19.50 | 0: 1.00 | 0: 1.00 |
| 7 | 0: 16.42 | 0: 1.00 | 0: 1.00 |
| 8 | 0: 16.40 | 0: 1.00 | 0: 1.00 |
| 9 | 0: 14.88 | 0: 0.99 | 0: 1.00 |
| 10 | 0: 12.76 | 0: 0.99 | 0: 1.00 |
| 11 | 0: 9.76 | 0: 0.94 | 0: 0.986 |
| 12 | 0: 7.52 | 0: 0.98 | 0: 0.99 |
| Character 3, Subcrustose growth | | | |
| 1 | 0: 4.28 | 0: 0.99 | 0: 1.00 |
| 2 | Ambiguity | 1: 0.62 | 0: 1.00 |
| 3 | 0: 9.68 | 0: 1.00 | 0: 1.00 |
| 4 | 0: 8.82 | 0: 1.00 | 0: 1.00 |
| 5 | 0: 11.48 | 0: 1.00 | 0: 1.00 |
| 6 | 0: 13.60 | 0: 1.00 | 0: 1.00 |
| 7 | 0: 10.74 | 0: 1.00 | 0: 1.00 |
| 8 | 0: 10.70 | 0: 1.00 | 0: 1.00 |
| 9 | 0: 7.40 | 0: 1.00 | 0: 1.00 |
| 10 | 0: 3.24 | 0: 0.49 | 0: 1.00 |
| 11 | 0: 4.74 | 0: 0.90 | 0: 0.99 |
| 12 | 0: 6.16 | 0: 1.00 | 0: 1.00 |
| Character 4, Crustose growth | | | |
| 1 | 0: 8.46 | 0: 1.00 | 0: 1.00 |
| 2 | Ambiguity | 0: 1.00 | 0: 1.00 |
| 3 | 0: 12.10 | 0: 1.00 | 0: 1.00 |
| 4 | 0: 11.25 | 0: 1.00 | 0: 1.00 |
| 5 | 0: 12.48 | 0: 1.00 | 0: 1.00 |
| 6 | 0: 14.72 | 0: 1.00 | 0: 1.00 |
| 7 | 0: 12.18 | 0: 1.00 | 0: 1.00 |
| 8 | 0: 12.34 | 0: 1.00 | 0: 1.00 |
| 9 | 0: 11.60 | 0: 1.00 | 0: 1.00 |
| 10 | 0: 9.44 | 0: 1.00 | 0: 1.00 |
| 11 | 0: 10.46 | 0: 1.00 | 0: 0.99 |
| 12 | 0: 11.36 | 0: 1.00 | 0: 1.00 |
| Character 5, Umbilicate growth | | | |
| 1 | 0: 8.58 | 0: 1.00 | 0: 1.00 |
| 2 | Ambiguity | 0: 0.99 | 0: 1.00 |
| 3 | 0: 8.80 | 0: 1.00 | 0: 1.00 |
| 4 | 0: 8.42 | 0: 1.00 | 0: 1.00 |
| 5 | 0: 8.84 | 0: 1.00 | 0: 1.00 |
| 6 | 0: 9.20 | 0: 1.00 | 0: 1.00 |
| 7 | 0: 8.88 | 0: 1.00 | 0: 1.00 |
| 8 | 0: 8.96 | 0: 1.00 | 0: 1.00 |
| 9 | 0: 8.62 | 0: 1.00 | 0: 1.00 |
| 10 | 0: 8.52 | 0: 1.00 | 0: 1.00 |
| 11 | 0: 8.60 | 0: 1.00 | 0: 0.99 |
| 12 | 0: 8.72 | 0: 1.00 | 0: 1.00 |
| Character 6, Epicortex present | | | |
| 1 | 1: 23.42 | 1: 1.00 | 1: 1.00 |
| 2 | 1: 24.26 | 1: 1.00 | 1: 1.00 |
| 3 | 1: 22.71 | 1: 1.00 | 1: 1.00 |
| 4 | 1: 19.90 | 1: 1.00 | 1: 1.00 |
| 5 | 1: 24.09 | 1: 1.00 | 1: 1.00 |
| 6 | 1: 24.34 | 1: 1.00 | 1: 1.00 |
| 7 | 1: 21.29 | 1: 1.00 | 1: 1.00 |
| 8 | 1: 21.26 | 1: 1.00 | 1: 1.00 |
| 9 | 1: 26.67 | 1: 1.00 | 1: 1.00 |
| 10 | 1: 24.97 | 1: 1.00 | 1: 1.00 |
| 11 | 1: 24.86 | 1: 1.00 | 1: 0.99 |
| 12 | 1: 29.23 | 1: 1.00 | 1: 1.00 |
| Character 7, Epicortex pored | | | |
| 1 | 0: 2.87 | 0: 0.29 | 1: 0.712 |
| 2 | 1: 8.75 | 1: 1.00 | 1: 1.00 |
| 3 | 0: 21.92 | 0: 1.00 | 0: 1.00 |
| 4 | 1: 5.60 | 1: 0.78 | 1: 1.00 |
| 5 | 1: 15.66 | 1: 1.00 | 1: 1.00 |
| 6 | 1: 14.98 | 1: 1.00 | 1: 1.00 |
| 7 | 0: 18.15 | 0: 1.00 | 0: 1.00 |
| 8 | 0: 22.39 | 0: 1.00 | 0: 1.00 |
| 9 | 0: 24.06 | 0: 1.00 | 0: 1.00 |
| 10 | 0: 2.94 | 0: 0.28 | 1: 0.712 |
| 11 | 0: 10.99 | 0: 1.00 | 0: 0.99 |
| 12 | 0: 21.84 | 0: 1.00 | 0: 1.00 |
| Character 8, Epicortex non-pored | | | |
| 1 | 1: 4.26 | 1: 0.46 | 0: 0.911 |
| 2 | 0: 7.88 | 0: 0.99 | 0: 1.00 |
| 3 | 1: 22.46 | 1: 1.00 | 1: 1.00 |
| 4 | 0: 4.12 | 0: 0.67 | 0: 1.00 |
| 5 | 0: 13.82 | 0: 1.00 | 0: 1.00 |
| 6 | 0: 13.22 | 0: 1.00 | 0: 1.00 |
| 7 | 1: 18.58 | 1: 1.00 | 1: 1.00 |
| 8 | 1: 21.90 | 1: 1.00 | 1: 1.00 |
| 9 | 1: 24.56 | 1: 1.00 | 1: 1.00 |
| 10 | 1: 4.40 | 1: 0.46 | 0: 1.00 |
| 11 | 1: 12.22 | 1: 1.00 | 1: 0.99 |
| 12 | 1: 23.22 | 1: 1.00 | 1: 1.00 |
| Character 9, Pseudocyphellae | | | |
| 1 | 1: 6.58 | 1: 0.95 | 0: 1.00 |
| 2 | 0: 6.51 | 0: 0.99 | 0: 1.00 |
| 3 | 1: 21.04 | 1: 1.00 | 1: 1.00 |
| 4 | 0: 1.61 | 0: 0.07 | 0: 1.00 |
| 5 | 0: 10.74 | 0: 1.00 | 0: 1.00 |
| 6 | 0: 10.45 | 0: 1.00 | 0: 1.00 |
| 7 | 1: 18.17 | 1: 1.00 | 1: 1.00 |
| 8 | 1: 19.55 | 1: 1.00 | 1: 1.00 |
| 9 | 1: 23.18 | 1: 1.00 | 1: 0.585 |
| 10 | 1: 6.82 | 1: 0.95 | 0: 1.00 |
| 11 | 1: 13.85 | 1: 1.00 | 1: 0.215 |
| 12 | 1: 20.62 | 1: 1.00 | 0: 0.383 |
| Character 10, Atranorin | | | |
| 1 | 0: 3.99 | 0: 0.78 | 1: 0.071 |
| 2 | Ambiguity | 0: 1.00 | 0: 1.00 |
| 3 | 1: 8.53 | 1: 1.00 | 1: 1.00 |
| 4 | 1: 8.16 | 1: 1.00 | 1: 1.00 |
| 5 | 1: 16.09 | 1: 1.00 | 1: 1.00 |
| 6 | Ambiguity | 1: 0.32 | 1: 1.00 |
| 7 | 1: 12.27 | 1: 1.00 | 1: 1.00 |
| 8 | 1: 10.44 | 1: 1.00 | 1: 1.00 |
| 9 | 0: 10.60 | 0: 1.00 | 0: 1.00 |
| 10 | Ambiguity | 0: 0.83 | 0: 0.380 |
| 11 | Ambiguity | 0: 0.76 | 0: 0.525 |
| 12 | Ambiguity | 0: 0.69 | 0: 0.532 |
| Character 11, Usnic acid | | | |
| 1 | 1: 5.62 | 1: 0.89 | 0: 0.945 |
| 2 | Ambiguity | 1: 0.90 | 0: 0.489 |
| 3 | 0: 6.01 | 0: 0.99 | 0: 1.00 |
| 4 | 0: 2.02 | 0: 0.18 | 0: 1.00 |
| 5 | 0: 9.06 | 0: 1.00 | 0: 1.00 |
| 6 | Ambiguity | 1: 0.18 | 0: 1.00 |
| 7 | 0: 6.28 | 0: 0.99 | 0: 1.00 |
| 8 | 0: 5.66 | 0: 0.97 | 0: 1.00 |
| 9 | 0: 2.60 | 0: 0.20 | 0: 1.00 |
| 10 | Ambiguity | 1: 0.88 | 0: 0.614 |
| 11 | Ambiguity | 1: 0.70 | 0: 0.99 |
| 12 | Ambiguity | 0: 0.56 | 0: 1.00 |
| Character 12, Melanin | | | |
| 1 | 0: 1.35 | 0: 1.00 | 0: 1.00 |
| 2 | Ambiguity | 0: 0.47 | 0: 1.00 |
| 3 | 0: 6.96 | 0: 1.00 | 0: 1.00 |
| 4 | 0: 7.58 | 1: 1.00 | 0: 1.00 |
| 5 | 0: 15.50 | 0: 1.00 | 0: 1.00 |
| 6 | Ambiguity | 0: 1.00 | 0: 1.00 |
| 7 | 0: 11.00 | 0: 1.00 | 0: 1.00 |
| 8 | 0: 9.68 | 0: 1.00 | 0: 1.00 |
| 9 | 1: 12.37 | 1: 0.99 | 1: 1.00 |
| 10 | Ambiguity | 0: 0.59 | 0: 1.00 |
| 11 | Ambiguity | 0: 0.56 | 0: 1.00 |
| 12 | Ambiguity | 0: 0.53 | 0: 1.00 |
